# sEV-mediated lipid droplets transferred from bone marrow adipocytes promote ferroptosis and impair osteoblast function

**DOI:** 10.1016/j.jlr.2024.100657

**Published:** 2024-09-24

**Authors:** Weibo Huang, Feng Hua, Tong Su, Chenghao Zhou, Kangcheng Zhao, Dianwen Song

**Affiliations:** 1 Department of Orthopedics, Shanghai General Hospital, Shanghai Jiao Tong University School of Medicine, Shanghai, China; 2 Department of Orthopedics, The First Affiliated Hospital, Zhejiang University School of Medicine, Hangzhou, China

**Keywords:** Osteoporosis, Bone marrow adipocyte, ferroptosis, osteoblast, lipid droplet

## Abstract

Osteoporosis is linked to increased bone marrow adipocyte (BMAd) proliferation, which displaces bone-forming cells and alters the local environment. The impact of BMAd lipid droplets on bone health and osteoblast function remains unclear. This study investigates the interplay between BMAd-derived lipid droplets and osteoblast functionality, focusing on ferroptosis pathways. Osteoblast cultures were treated with conditioned media from adipocytes to simulate in vivo conditions. High-throughput mRNA sequencing and Western blot analysis were used to profile changes in gene expression and protein levels related to ferroptosis, oxidative phosphorylation, and osteogenic markers. Cellular assays assessed the direct impact of lipid droplets on osteoblast activity. Results showed that osteoblasts exposed to adipocyte-conditioned media had increased intracellular lipid droplet accumulation, upregulation of ferroptosis-related genes and proteins, and downregulation of oxidative phosphorylation and osteoblast differentiation markers. Treatment with ferroptosis inhibitors reversed the detrimental effects on osteoblasts, indicating the functional relevance of this pathway in osteoporosis. BMAd-derived lipid droplets contribute to osteoblast dysfunction through ferroptosis induction. Inhibiting ferroptosis could preserve osteoblast function and combat osteoporosis-related bone issues, suggesting that modulating lipid metabolism and redox balance in bone cells may be promising for future treatments.

Bone marrow adipocytes (BMAds) constitute 10% of total body fat and occupy 50%–70% of the marrow cavity space (1, 2). Historically viewed merely as fillers, BMAds received scant attention compared to other marrow cells. However, increasing studies revealed the roles of BMAds in the bone marrow microenvironment under physiological and pathological conditions. Increased BMAds abundance could be commonly observed in bone loss caused by various factors such as aging, postmenopausal period, obesity, as well as the use of radiotherapy, chemotherapy, and glucocorticoids (3). These factors may lead to some pathophysiological changes such as the shift in differentiation of bone marrow mesenchymal stem cells (BMSCs) from osteoblasts to BMAds and adipocyte infiltration, resulting in bone marrow adiposity (4, 5, 6). Some reports have proven that BMAds play a role in the bone marrow environment and potentially affect other cells (7, 8). Although the secretory function of bone marrow adipocytes and their role in the cellular signaling of osteoblasts remain significant, questions persist regarding their overall impact and their effects on osteoblasts during the aging process.

Research has indicated that BMAds may influence the bone marrow microenvironment through cell-to-cell contact, release of extracellular vesicles (EVs), and secretion of adipokines and cytokines (9, 10, 11). Small extracellular vesicles (sEVs), tiny carriers released by all cell types, are of great interest for their role in osteoporosis (12, 13, 14). In osteoporosis, sEVs transport bioactive molecules like lipids, proteins, and nucleic acids between bone marrow adipocytes and osteoblastic cells (15, 16, 17). This exchange actively regulates cellular processes, affecting bone metabolism and potentially worsening the disease (18, 19).

Previous literature has revealed that extracellular vesicles can transfer certain organelles, such as mitochondria, facilitating intercellular communication (17). The interplay between adipose storage and osteoblasts involves metabolic cross-talk. The metabolism of lipid droplets in adipocytes may impede the function and viability of adjacent osteoblasts, with past studies reporting the potential transfer of lipid droplets from adipocytes to surrounding cells (20). Indeed, the occurrence of abnormal lipid droplets in non-adipogenic lineage cells is not a novel concept—for instance, lipid storage has been extensively studied in hepatocytes. Long-term alcohol consumption leads to increased intracellular lipid deposition in bone cells, characterized by the appearance of lipid droplets, which typically precede osteoblast apoptosis and bone necrosis (21). During rapid bone tissue remodeling, lipids in bone marrow adipocytes can serve as an energy source for osteoblasts. However, lipolysis of lipid droplets generates free fatty acids that may exert lipotoxic effects, impacting osteoblasts through the induction of autophagy and apoptosis mechanisms, ultimately contributing to long-term bone loss (22).

In this study, we discovered abnormal lipid droplets in osteoblasts through bone tissue specimens from patients with osteoporosis. We found that these abnormal lipid droplets may originate from bone marrow adipocytes through exosome delivery. Upon entry into osteoblasts, the expression of ABHD5 is upregulated, which is a crucial activator of intracellular lipolysis. Subsequently, this leads to an upregulation of lipolysis and an increase in intracellular polyunsaturated fatty acids (PUFA). Consequently, this induces ferroptosis in osteoblasts, ultimately leading to osteoporosis. This study aims to explore the potential impacts and specific mechanisms of bone marrow adipocytes on osteoblasts, to elucidate their exact roles in the occurrence and development of osteoporosis, and to identify potential intervention targets, thereby providing more clues and strategies for the prevention and treatment of this disease.

## Materials and Methods

### Animals

Female C57BL/6J mice aged six weeks were procured from the animal facility at Shanghai Jiao Tong University. All procedures involving animal studies were previously approved by the institutional review board of Shanghai General Hospital, Shanghai Jiaotong University, Shanghai (No.2024AWS254). Prior to the commencement of the experimental protocol, the mice underwent a one-week acclimatization period under specific pathogen-free (SPF) conditions. They were housed in cages with controlled temperatures (23–25°C), a 12-h light-dark cycle (lights on from 6:00 to 18:00), and a humidity range of 50%–70%. Mice were provided with standard rodent chow and water ad libitum throughout the acclimatization and experimental periods. To emulate postmenopausal osteoporosis, ovariectomies (OVX) were conducted on the mice under aseptic conditions following ethical approval. Following OVX, mice were randomly assigned to experimental groups ensuring equal distribution. To mimic the metabolic milieu of obesity, OVX mice were subsequently placed on a high-fat diet (HFD) consisting of 21% fat and 0.15% cholesterol for 12 consecutive weeks. After the 12-week dietary intervention, all mice were euthanized, and bone tissues were carefully harvested for comprehensive analysis. To evaluate the impact of obesity on bone health, bone morphometric parameters were assessed using micro-computed tomography (μCT) assays.

### Cell culture

Mesenchymal stem cells (MSCs) were isolated from the bone marrow aspirates of adult mice. Firstly, the femurs and tibias were aseptically dissected, and bone marrow was flushed out using a sterile syringe filled with Dulbecco's Modified Eagle Medium (DMEM) supplemented with 10% fetal bovine serum (FBS) and 1% penicillin-streptomycin solution. The cell suspension was then passed through a 70 μm cell strainer to remove debris and clumps. The filtrate was centrifuged at 1,200 rpm for 5 min at 4°C, and the supernatant was discarded. The resulting cell pellet was resuspended and treated with ACK lysis buffer (0.15 M NH4Cl, 10 mM KHCO3, and 0.1 mM EDTA, pH 7.2–7.4) for 5 min at room temperature to lyse red blood cells. After another centrifugation step at 1,200 rpm for 5 min, the cell pellet was washed twice with PBS and re-suspended in a complete culture medium (DMEM with 10% FBS, 1% penicillin-streptomycin, and 2 mM L-glutamine). The isolated cells were seeded in T75 flasks at a density of approximately 1 × 10ˆ6 cells/cm^2^ and incubated at 37°C in a humidified atmosphere containing 5% CO2. Non-adherent cells were removed by changing the culture medium 48 h post-seeding. Upon reaching 70%–80% confluence, adherent cells were detached using 0.25% trypsin-EDTA solution, counted, and passaged at a ratio of 1:3. This process was repeated until a homogeneous population of MSCs was established, typically after two to three passages. For differentiation assays, confluent MSCs were plated in 6-well plates and cultured in adipogenic (abs9801, absin) or osteogenic (abs9800, absin) induction media, as recommended by the manufacturer. The differentiation process was initiated by replacing the culture medium with the respective induction medium and maintained for 10 days. Media were changed every 3 days. During the differentiation period, adipogenesis was verified by bodily staining for lipid droplets, while osteogenesis was confirmed by Alizarin Red S staining for calcium deposits.

### Clinical specimens

During orthopedic surgical interventions necessitated by traumatic fractures, segments of cancellous and cortical bone tissue were selectively harvested from easily accessible areas in patients undergoing surgery at Shanghai General Hospital. Patients enrolled in the study were selected based on predefined inclusion criteria, which encompassed age, gender, absence of pre-existing bone diseases, and no history of medications affecting bone metabolism. The patients' body mass index (BMI) was meticulously documented to assess obesity status according to World Health Organization (WHO) classification standards. Additionally, pertinent medical records were reviewed to collect demographic information, fracture etiology, laboratory test results, and other clinical parameters relevant to bone health assessment. Prior to the surgical procedure and specimen collection, informed consent was obtained from each patient, detailing the purpose of the study, the use of collected material, and the assurance of anonymity and confidentiality in handling personal data. This study strictly adhered to the principles outlined in the Declaration of Helsinki and received full ethical clearance from the Institutional Review Board of Shanghai General Hospital (Ethical Review Number:No. 2024SQ232). Following surgical extraction, bone tissue specimens were immediately immersed in 4% paraformaldehyde solution buffered to a pH of 7.4 for fixation, ensuring thorough penetration. The duration of fixation was standardized to a minimum of 24 h to ensure adequate preservation of cellular morphology and antigenicity. Post-fixation, the bone samples were thoroughly rinsed with phosphate-buffered saline (PBS) and stored in 70% ethanol at 4°C until further processing. The specimens were processed for decalcification, dehydration, paraffin embedding, and sectioning at a thickness of 4 μm. Quality control measures were implemented to verify the integrity of the samples and ensure consistency across all stages of preparation.

### Isolation of lipid droplets

Lipid droplets were isolated according to the isolation kit (MET5011, CELLBIOLABS). The protocol was started by trypsinizing approximately 5 × 10ˆ7 adipocytes, which were then resuspended in 10 ml of growth media within a 15 ml polypropylene tube. The cells were centrifuged at 1,000*g* for 5 min to pellet. The media was aspirated, and the cells were washed twice with 10 ml of PBS, each wash was followed by pelleting and media removal. After the final wash, the cells were resuspended in PBS, transferred to a 2 ml microcentrifuge tube, and the centrifugation step was repeated. The PBS was aspirated, and the pellet was resuspended in 200 μl of Reagent A and incubated on ice for 10 min. Next, 800 μl of Reagent B was introduced, ensuring thorough mixing before another 10-min ice incubation. The cells were then homogenized by passing the mixture through a 27-gauge needle five times. A short spin was conducted, after which 600 μl of Reagent B was gently layered over the homogenate. The sample was subjected to a lengthy 3-h centrifugation at 20000 *g* and 4°C. Finally, 270 μl of the supernatant, containing the floating lipid droplets, was carefully withdrawn and transferred into a new microcentrifuge tube for further processing.

### Immunofluorescence

Freshly excised bone tissues were immersed in a 10% ethylene diamine tetraacetic acid (EDTA) decalcification solution, which was refreshed every 2–3 days until complete decalcification was achieved as assessed by radiographic transparency and/or histological examination, typically lasting for 2 weeks. Decalcified tissues were embedded in paraffin wax and sectioned at 4 μm thickness using a rotary microtome. The sections were dewaxed in xylene and rehydrated through a graded series of ethanol solutions, finishing in distilled water. Heat-induced epitope retrieval was performed using citrate buffer (pH 6.0) in a microwave oven at 95°C for 15 min. The sections were blocked with 10% goat serum for 1 h at room temperature to reduce nonspecific binding, followed by incubation with the primary antibody diluted in blocking buffer overnight at 4°C. The primary antibodies used were directed against target antigens including PLIN1(ab3526, Abcam) or OPN (ab63856, Abcam) at a dilution of 1:200. After incubation, the sections were washed thrice with PBS-Tween (0.1%) for 5 min each to remove unbound primary antibodies. Sections were then incubated with Donkey Anti-Rabbit IgG H&L (Alexa Fluor® 647) (ab150075, Abcam) or Goat Anti-Rabbit IgG H&L (Alexa Fluor® 488) (ab150077, Abcam) for 1 h at room temperature in the dark. The secondary antibodies were diluted 1:500 in PBS. Nuclei were counterstained with 4′,6-diamidino-2-phenylindole (DAPI) for 5 min. Slides were then rinsed, air-dried, and mounted with an antifade mounting medium. Cells were grown on glass coverslips, fixed with 4% paraformaldehyde for 15 min at room temperature, and permeabilized with 0.1% Triton X-100 in PBS for 10 min. After washing, cells were blocked with 10% goat serum in PBS for 1 h and incubated with the same primary antibodies as mentioned above overnight at 4°C. Similar to the tissue protocol, cells were washed, incubated with species-appropriate secondary antibodies, and then counterstained with DAPI. As for the fluorescent dye including Bodipy (C2053S, Beyotime), Bodipy-C11 (27,086-1, Cayman Chemical), JC1 (C2005, Beyotime), and Mitotracker (C1035, Beyotime), the cells were incubated with them for 30 min. Fluorescence images were captured using a Zeiss LSM 880 confocal microscope equipped with the appropriate filters for the respective fluorophores. Image acquisition settings were kept constant across comparable samples to allow for valid comparison.

### Flow cytometry

Cells were first harvested from the culture flask by gentle trypsinization, pelleted by centrifugation at 300 *g* for 5 min at room temperature, and resuspended in phosphate-buffered saline (PBS) without calcium or magnesium. To eliminate residual culture medium and cellular debris, cells were washed thrice with cold PBS centrifuged at 300 *g* for 5 min each time. Post-washing, the cell pellets were fixed using a predetermined volume of 4% paraformaldehyde solution for 15 min at room temperature, followed by permeabilization with ice-cold 90% methanol for 30 min on ice. After permeabilization, cells were washed twice with PBS containing 2% FBS. The fixed and permeabilized cells were then resuspended in blocking buffer (5% FBS and 0.5% Tween-20 in PBS) and incubated for 30 min at room temperature to block non-specific binding sites. Subsequently, cells were incubated with primary antibodies OPN(CL488-22952, PROTEINTECH) or RUNX2(CL488-20700, PROTEINTECH) or PLIN1(ab3526, Abcam) for 1 h at room temperature in the dark, gently shaking. Following incubation with primary antibodies, cells were washed three times with PBS to remove unbound primary antibodies. Next, the cells were incubated with Bodipy (C2053S, Beyotime) or Donkey Anti-Rabbit IgG H&L (Alexa Fluor® 647) (ab150075, Abcam) for 45 min at room temperature in the dark, with gentle agitation. After secondary antibody staining, cells were washed three more times with PBS to eliminate excess secondary antibodies. Prepared cells were resuspended in PBS and analyzed using a BD FACSCanto™ II flow cytometer for the detected fluorophores. Collected data were analyzed using FlowJo software. Gates were set based on unstained controls to define the boundaries of positive and negative populations. To perform flow cytometry detection of Bodipy-stained exosomes, we resuspended the isolated exosomes in a buffer containing Bodipy dye and incubated the mixture at room temperature for 1 h. Following incubation, the exosomes were separated again. The exosomes were then diluted in PBS and analyzed by flow cytometry to detect Bodipy staining in the exosomes.

### Exosome isolation and identification

To isolate exosomes utilizing Invitrogen™ Dynabeads™ Protein G (Cat. No. 10003D), the following protocol was adhered to, in accordance with the manufacturer's instructions. Initially, the cell culture supernatant was collected and subjected to centrifugation to eliminate cellular debris and larger particulate matter. Subsequently, the clarified supernatant was combined with exosome-specific antibodies and magnetic beads provided within the kit. The mixture was then incubated at an appropriate temperature for an extended period to facilitate the efficient binding of antibodies to the exosomal surface markers. Unbound components were removed via magnetic separation, and the magnetic beads were extensively washed with an appropriate buffer to remove non-specifically bound proteins and other contaminants. Finally, the exosomes were eluted from the magnetic beads for further analysis. Isolated exosomes were characterized for size distribution and morphology using nanoparticle tracking analysis (NTA) and transmission electron microscopy (TEM), respectively. Additionally, their identity was confirmed by Western blot analysis, probing for specific exosomal markers like flotillin and CD81. Throughout the procedure, strict aseptic techniques were maintained to ensure the absence of contamination.

### Western blot analysis

Western Blot Analysis Cells were lysed in RIPA buffer supplemented with protease inhibitors (PMSF). Total protein concentrations were measured using a BCA protein assay kit to ensure equal loading. An aliquot containing equal amounts of protein was separated by SDS-PAGE on a 10% gel and transferred onto PVDF membranes. After transfer, membranes were blocked with 5% non-fat milk in TBS-Tween for 1 h at room temperature. Primary antibodies targeting specific proteins including flotillin (ab133497, Abcam), CD81 (ab219209, Abcam), PLIN1 (ab317260, Abcam), PLIN2 (ab270274, Abcam), OPN (ab283656, Abcam), RUNX2 (ab236639, Abcam), OCN (ab93876, Abcam), GPX4 (ab125066, Abcam), TFRC (ab214039, Abcam), ABHD5 (12201-1-AP, Proteintech), GAPDH (ab9485, Abcam), β-Actin (ab8227, Abcam), and ATGL (ab207799, Abcam) were incubated overnight at 4°C. Following three washes with TBS-Tween, membranes were incubated with Goat Anti-Rabbit IgG H&L (HRP) (ab6721, Abcam) for 1 h at room temperature. Proteins were detected using an enhanced chemiluminescence substrate and imaged using a ChemiDoc imaging system. Bands were quantified densitometrically with ImageJ software. As a loading control, blots were probed with an antibody against GAPDH or β-Actin.

### Alizarin red S staining

The cells were fixed with 4% paraformaldehyde solution for 15 min at room temperature. After fixation, the cells were rinsed several times with PBS to remove excess fixative. Next, the cells were incubated with a 2% Alizarin Red S staining solution (adjusted to pH 4.2) for 10 min at room temperature with gentle shaking. The stained cells were then washed with PBS thrice for 5 min each to remove unbound dye. Finally, mineralized nodules were visualized under an inverted microscope using bright-field illumination at a specific magnification. For quantitative analysis, images were captured and analyzed using a dedicated image analysis software. Note that pre-treatment with an acidic solution might be necessary depending on the experimental context.

### High-throughput mRNA sequencing and analysis

High-Throughput mRNA Sequencing and Analysis Total RNA was isolated from cells using Trizol reagent following a mechanical disruption. After DNase I treatment to remove genomic DNA contamination, RNA integrity, and concentration were assessed using both a NanoDrop spectrophotometer and an Agilent Bioanalyzer, with a minimum RNA Integrity Number (RIN) of 7.0 accepted for library preparation. mRNA libraries were constructed using poly(A) selection and sequenced on the Illumina HiSeq platform (paired-end 150 bp reads), as provided by NOVELbio. Transcript abundance estimation and differential expression analysis were performed with the DESeq2 package in R, setting a |log2 fold change| > 1 and adjusted *P*-value < 0.05 as the criteria for identifying differentially expressed mRNAs. Subsequently, the identified differentially expressed mRNAs were subjected to Kyoto Encyclopedia of Genes and Genomes (KEGG) pathway enrichment analysis, with a false discovery rate (FDR) adjusted *P*-value < 0.05 considered statistically significant. Moreover, Gene Set Enrichment Analysis (GSEA) was conducted on the normalized mRNA expression data to determine whether predefined gene sets were enriched among the differentially expressed genes using the MSigDB database. Gene sets with an FDR q-value < 0.05 and a normalized enrichment score (NES) > 1 were considered significantly enriched.

### Seahorse metabolism analysis

Specifically, cells were seeded at a density of 5 × 10ˆ5 cells/well onto Seahorse XF96 cell culture microplates and allowed to attach and proliferate overnight at 37°C under a humidified atmosphere with 5% CO2. Before commencing the assay, the growth medium was exchanged for Seahorse XF base medium, devoid of serum and specifically supplemented with glucose, glutamine, and pyruvate according to the manufacturer's instructions. The plates were then incubated at ambient oxygen levels for 1 h to ensure equilibration. Utilizing the Seahorse XF96 Extracellular Flux Analyzer (Agilent Technologies), we focused on the measurement of oxygen consumption rate (OCR) to evaluate mitochondrial respiration. This protocol included programmed injections of oligomycin to determine ATP production-dependent OCR, followed by FCCP to estimate the maximal respiratory capacity, and finally, rotenone plus antimycin A to assess non-mitochondrial OCR. During the assay, basal and maximal OCR were recorded.

### Bioinformatics molecular docking

Molecular docking studies were carried out using AutoDock Vina (version 1.2.5), with thorough preparation of both protein targets and small molecule ligands. Protein structures were obtained from the Protein Data Bank and underwent preparation using PDB2PQR for protonation states and charge assignment. The binding site was defined based on literature evidence and visual inspection using PyMOL, focusing on previously reported active sites or conserved pockets. Ligands were retrieved from PubChem and subjected to energy minimization using the MMFF94 force field in OpenBabel. Docking simulations were set up with a grid box centered on the defined binding pocket, with grid spacing optimized for the protein-ligand complex. The default scoring function of AutoDock Vina was employed to rank the docked conformations. Post-processing included the selection of poses based on binding affinity and visually inspecting them for plausible interactions within the binding site.

### Co-immunoprecipitation

To explore the interaction between PLIN1 and ABHD5, co-immunoprecipitation was conducted. Cells were lysed in RIPA buffer (50 mM Tris-HCl pH 7.4, 150 mM NaCl, 1% NP-40, 0.5% sodium deoxycholate, 0.1% SDS, 1 mM EDTA, protease, and phosphatase inhibitor cocktails) on ice for 30 min, followed by centrifugation at 14,000 rpm for 15 min to clarify the lysate. For immunoprecipitation, protein A/G agarose beads were preincubated with either anti-PLIN1 (ab316115, Abcam) or anti-ABHD5 (Cat No. 12201-1-AP, Proteintech) antibodies overnight at 4°C with gentle rotation. After extensive washing with lysis buffer (four times, 10 min each), bound complexes were eluted in SDS sample buffer with heating at 95°C for 5 min. As a control, a nonspecific IgG antibody was also used for IP. The eluted proteins were analyzed by Western blotting using primary antibodies against PLIN1 and ABHD5 or ATGL (ab207799, Abcam).

### Micro-computerized tomography (μCT) analysis

μCT scans were conducted using a Bruker μCT system (SkyScan 1,172 Micro-CT, Bruker MicroCT), adhering to the guidelines set forth by the American Society for Bone and Mineral Research. Prior to scanning, femur samples were fixed in 10% neutral buffered formalin, followed by dehydration in ascending grades of ethanol. Scanning parameters were optimized to achieve high-resolution images while minimizing radiation exposure: 60 kV voltage, 100 μA current, 926 ms exposure time, and an isotropic voxel size of 10 μm. For trabecular bone analysis, a 1.5 mm long Region of Interest (ROI) was defined, starting 0.5 mm proximal to the distal growth plate, to standardize the evaluation area. The cortical bone analysis included an assessment of the mid-diaphysis, with specific ROIs defined based on cortical thickness measurements. Reconstruction of 3D images and segmentation of bone from non-bone tissue were performed using NRecon and CTAn software (Bruker), respectively, with careful thresholding to differentiate bone from the background. A comprehensive suite of bone microarchitectural parameters was quantified, including but not limited to bone mineral density (BMD), bone volume/tissue volume (BV/TV), trabecular number (Tb.N), trabecular thickness (Tb.Th), trabecular pattern factor (Tb.Pf), total porosity, and bone surface/tissue volume ratio (BS/TV). All analyses were conducted blinded to group allocation, and scans were repeated in duplicate to ensure measurement reliability. These detailed assessments facilitated a thorough examination of the microstructural characteristics within both trabecular and cortical compartments of the femurs.

### Statistical analysis

In the statistical analysis of our data, all results are expressed as the mean ± standard error of the mean (SEM) to provide a measure of variability. The Student's *t* test was administered for comparison between the two groups. In cases involving multiple group comparisons, the one-way analysis of variance (ANOVA) was conducted, followed by appropriate post-hoc tests to pinpoint specific group differences. Throughout the analysis, *P*＜0.05 was considered statistically significant.

## Results

### Lipid droplet transfer from adipocytes to osteoblasts

**Fig. 1:**
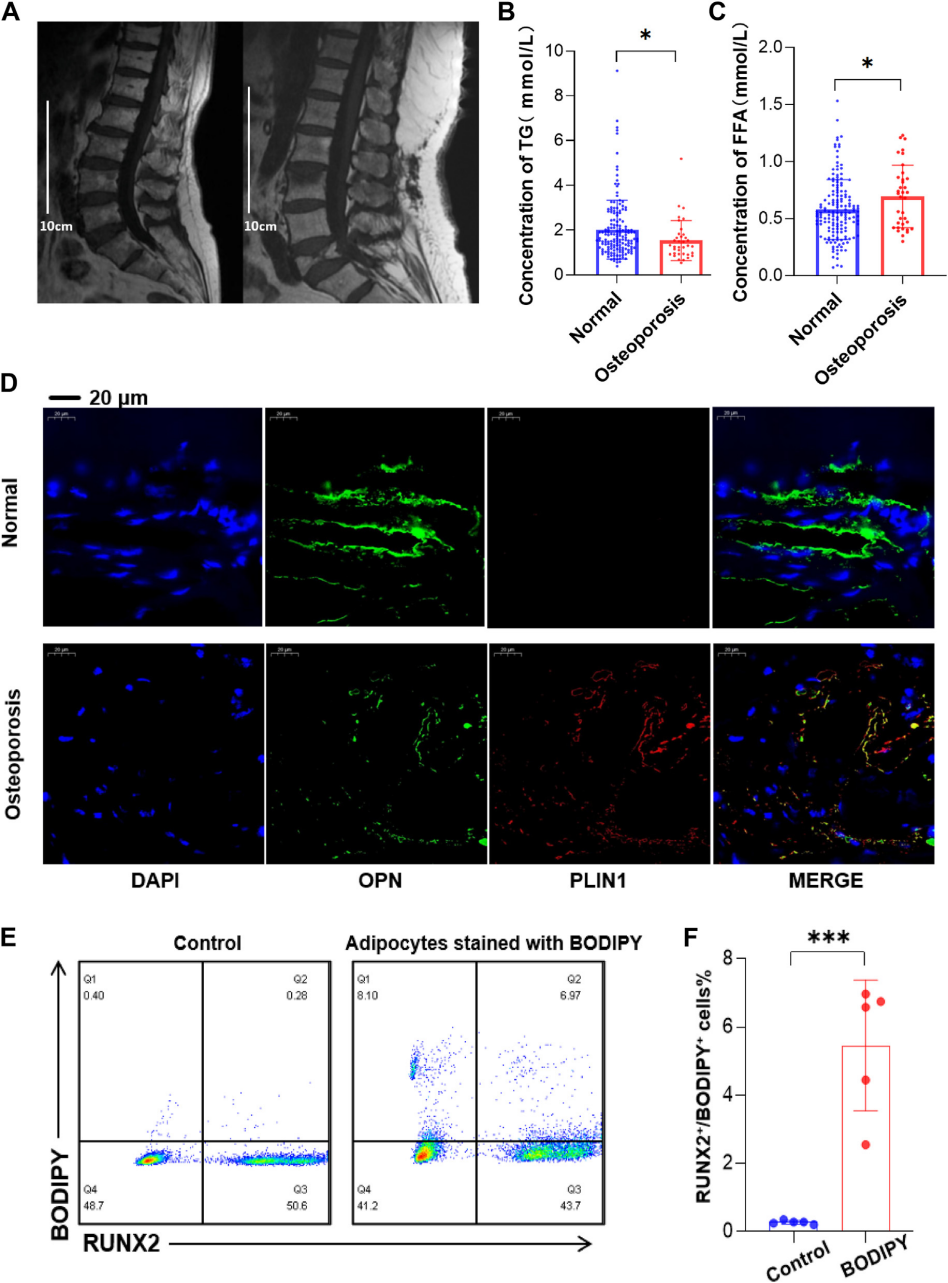
Increased bone marrow adipocytes were associated with osteoporosis, with adipocytes transferring lipid droplets to osteoblasts. A: The MRI T1 images of patients with osteoporotic vertebral compression fractures with low and high BMI are shown in the figure. Even in patients with low BMI, a large amount of high-signal adipose tissue can be scattered in the vertebral body, while in patients with high BMI, a uniform high-signal adipose area can be seen throughout the vertebral body, indicating that patients with low or high BMI can have an increase in bone marrow adipose tissue in the vertebral body. B and C: The laboratory examination found that the free fatty acid level was higher in osteoporosis patients while the triglyceride was lower in these patients. D: Osteopontin (OPN) and Perilipin 1 (PLIN1) fluorescent staining comparison in bone tissues from obese and normal-weight individuals. E and F: Following injection of pre-stained Bodipy-labeled adipocytes into mouse femurs, flow cytometry analysis was conducted to examine the co-localization of RUNX2 with Bodipy fluorescence.

The MRI T1 images of patients with osteoporotic vertebral compression fractures with low and high BMI are shown in Fig. 1A. These images reveal that a large amount of high-signal adipose tissue can be distributed in the vertebral body, indicating that patients with low or high BMI can have an increase in bone marrow adipose tissue in the vertebral body. According to the collected medical record of the osteoporosis patients (see Supplementary File), as Fig. 1B, C showed, the free fatty acid level was higher in osteoporosis patients while the triglyceride was lower in these patients, demonstrating the potentially different lipid metabolism pattern for osteoporosis patients. Moreover, multiple linear regression further confirmed that the TG and LDL-c levels were independently associated with osteoporosis. To elucidate the mechanisms by which obesity predisposes individuals to bone loss, we hypothesized that increasing adipocytes within the bone marrow may disrupt bone homeostasis. Immunofluorescence staining for PLIN1 (lipid droplet marker) and OPN (osteoblast marker) revealed PLIN1 expression in a subset of osteoblasts, indicating lipid droplets within these cells (Fig. 1D). To further investigate, we labeled 10^7^ adipocytes with Bodipy and transplanted them into the bone marrow of mice (n = 5 per group), with a separate group receiving a control injection. After 72 h, Bodipy fluorescence was observed in a portion of isolated osteoblasts, suggesting potential lipid uptake (Fig. 1E, F). These findings imply a potential transfer of lipid droplets from adipocytes to osteoblasts, a mechanism that could contribute to osteoporosis in obesity settings. Further mechanistic studies are warranted to confirm this hypothesis.

### Exosome-mediated lipid droplet transfer to osteoblasts

**Fig. 2:**
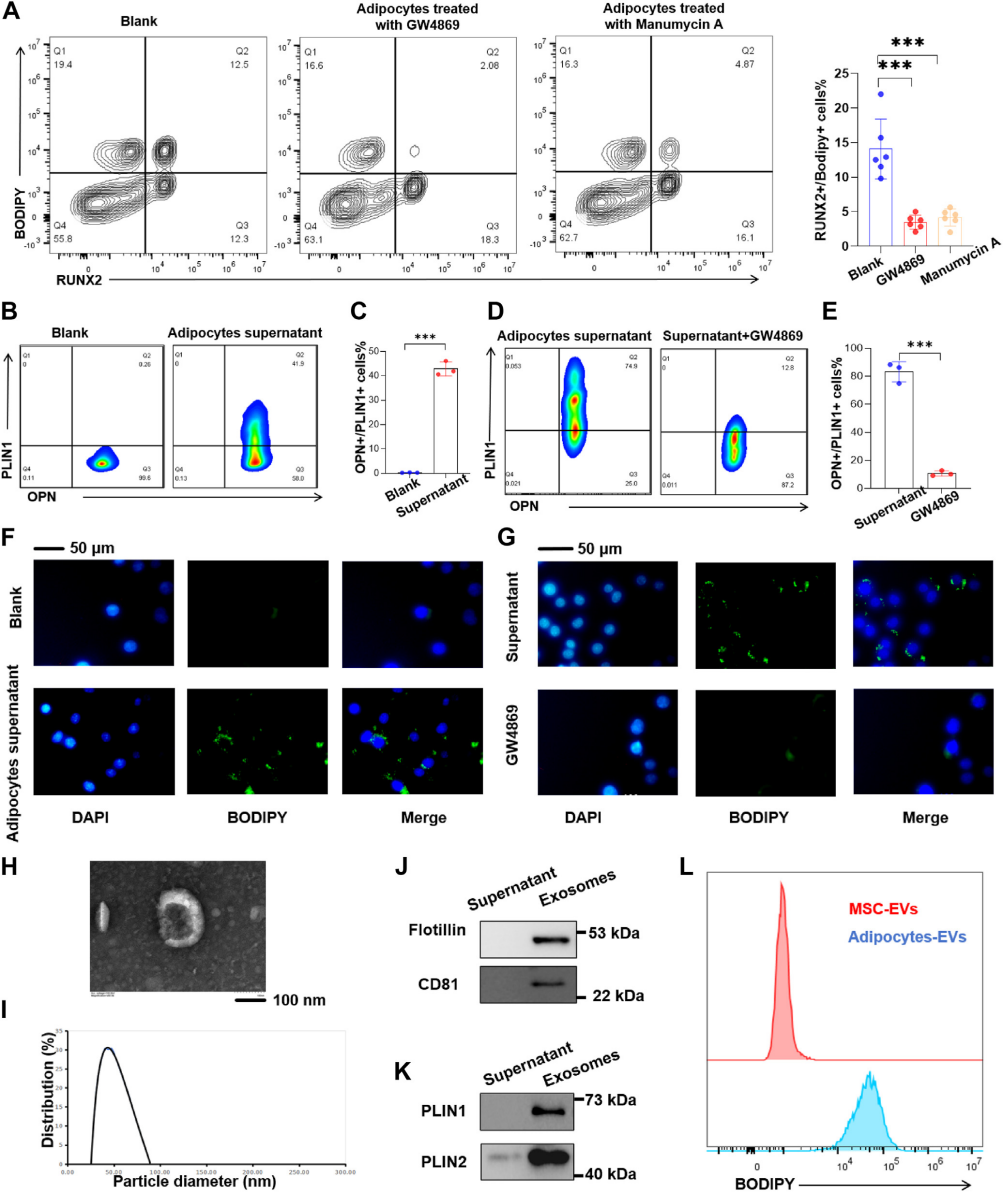
Adipocytes transferred lipid droplets to osteoblasts via small extracellular vesicles (sEVs). A: inhibiting exosome production in adipocytes using GW4869 or manumycin A, and then injecting these adipocytes into the bone marrow cavity, followed by flow cytometry to detect the expression levels of RUNX2 and bodipy in bone cells. B and C: supernatant from in vitro cultured adipocytes was added to osteoblast cultures, and flow cytometry assessed OPN and PLIN1 expression in osteoblasts. D and E: after GW4869 treatment, supernatant from adipocyte cultures was applied to osteoblasts, and flow cytometry examined OPN and PLIN1 levels. F: immunofluorescence detected Bodipy expression in osteoblasts following incubation with supernatant from adipocyte cultures. G: immunofluorescence visualized Bodipy in osteoblasts incubated with supernatant from GW4869-treated adipocytes. H: ultracentrifugation of adipocyte supernatant yields sEVs, which were examined under transmission electron microscopy for morphological characterization. (I) Nanoparticle tracking analysis determined the size distribution of sEVs isolated from adipocyte supernatant. J: Western blot analysis of sEV markers CD81 and Flotillin1 in sEVs isolated from adipocyte supernatant. K: Western blot quantified the content of lipid droplet markers PLIN1 and PLIN2 in the harvested sEVs. L: flow cytometry detection of Bodipy staining in mesenchymal stem cell exosomes and adipocyte exosomes.

To explore the mechanism of lipid droplet transfer, adipocytes were pre-treated with GW4869 or Manumycin A, both of which are inhibitors of exosome secretion, prior to injection. Quantitative analysis revealed a reduction in lipid droplet transfer to osteoblasts following GW4869/Manumycin A treatment (Fig. 2A). In vitro, osteoblasts co-cultured with adipocyte supernatant exhibited the presence of lipid droplets within them (Fig. 2B, C, F). Importantly, when adipocytes were pre-treated with GW4869 before supernatant harvest, subsequent co-culture with osteoblasts led to a decline in the number of osteoblasts acquiring lipid droplets (Fig. 2D, E, G), thereby reinforcing the role of exosomes. Exosomes were isolated from adipocyte supernatant via immunomagnetic beads and verified through transmission electron microscopy (Fig. 2H), Western blot analysis for exosomal markers CD81 and flotillin1 (Fig. 2J), and nanoparticle tracking analysis for exosome diameter measurement (Fig. 2I). Interestingly, these exosomes tested positive for lipid droplet markers (Fig. 2K) and BODIPY staining (Fig. 2L), indicating their capability to carry lipids. Collectively, these findings could support our hypothesis that exosomes derived from adipocytes mediate the transfer of lipid droplets to osteoblasts.

### Impairment of osteoblast function by lipid droplets

**Fig. 3:**
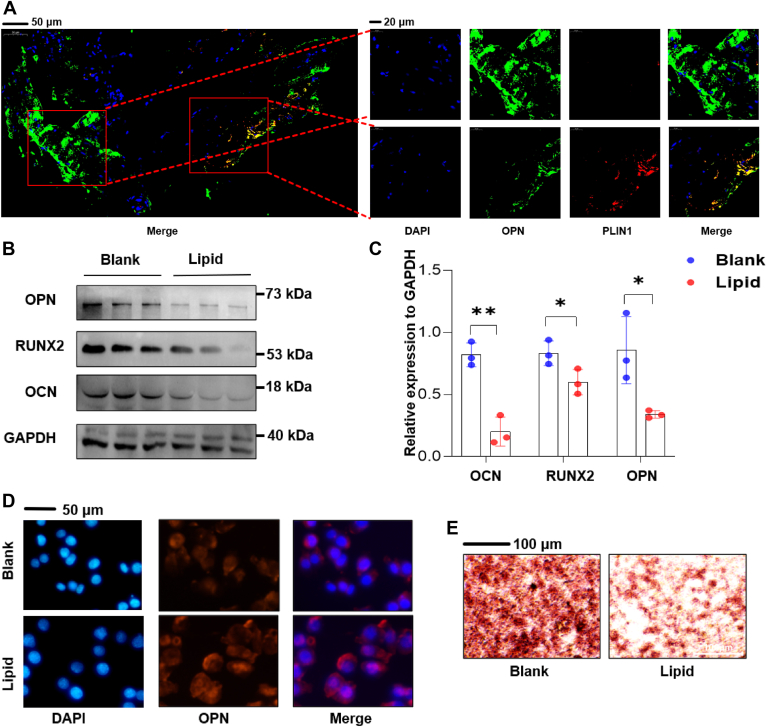
Osteoblasts with lipid droplet uptake exhibited impaired functionality. A: Immunofluorescent staining of Osteopontin (OPN) and Perilipin 1 (PLIN1) in bone tissue from the same patient. B and C: Western blot analysis of OPN, RUNX2, and Osteocalcin (OCN) expression levels in osteoblasts following incubation with lipid droplets. D: fluorescence detection of altered OPN expression in osteoblasts after lipid droplet treatment. E: Alizarin Red S staining to assess calcium nodule formation in osteoblasts upon lipid droplet treatment.

To assess the impact of lipid droplets on osteoblastic function, we conducted a comparative investigation using bone specimens derived from patients. Two distinct regions were meticulously chosen: one featuring osteoblasts that contained lipid droplets, and another where osteoblasts lacked these droplets. Our analysis demonstrated a decrease in the expression of osteopontin (OPN) within osteoblasts harboring lipid droplets compared to those without (Fig. 3A). To replicate these observations in vitro, we isolated lipid droplets and introduced them into the culture medium of osteoblasts. This experimental setup verified that osteoblasts treated with lipid droplets exhibited a reduction in the expression of multiple osteogenic proteins, thereby reinforcing the in vivo observations (Fig. 3B, C). Fluorescence found decreased OPN expression in osteoblasts exposed to lipid droplets (Fig. 3D). Additionally, alizarin red S staining revealed impaired mineralization in osteoblasts treated with lipid droplets (Fig. 3E). Collectively, these findings indicate an adverse effect of lipid droplets on the bone matrix deposition mediated by osteoblasts and their overall osteogenic potential.

### Lipid droplets modulate aerobic respiration and ferroptosis in osteoblasts

**Fig. 4:**
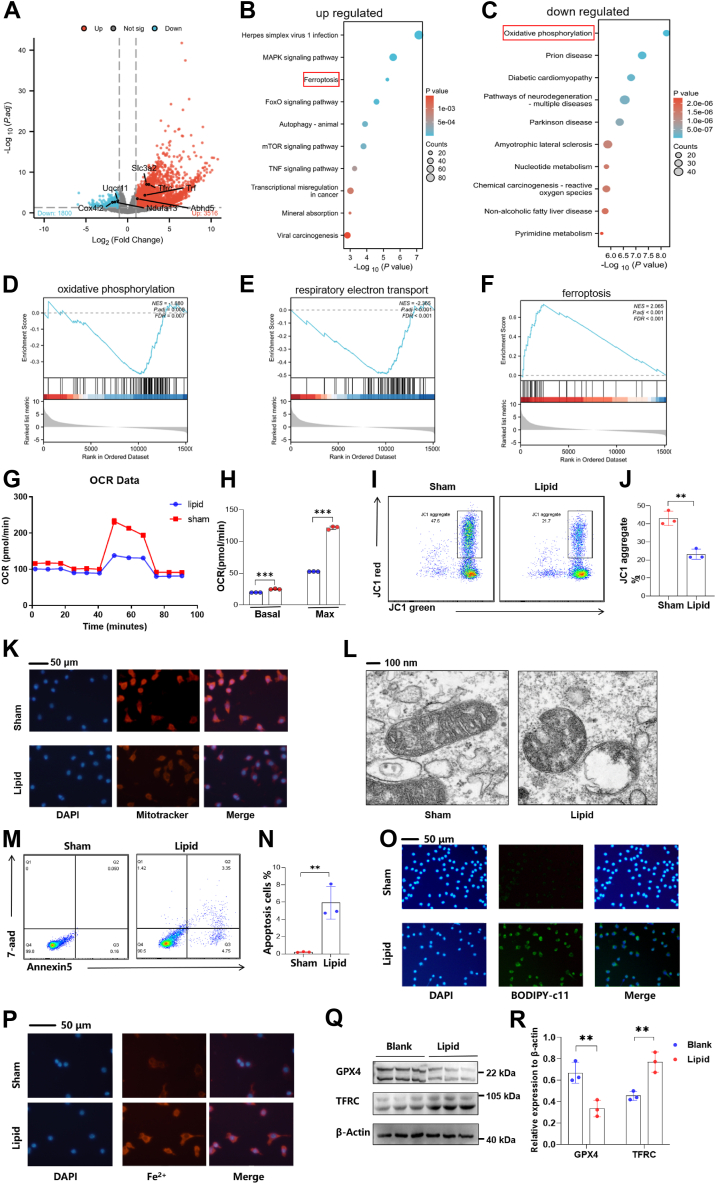
Lipid Droplet-Induced Mitochondrial Dysfunction and Ferroptosis in Osteoblasts. A: Volcano plot depicting differentially expressed genes after lipid droplet treatment in osteoblasts, filtered by *P* < 0.05 and |log2FC| > 1. B: Top ten enriched pathways among upregulated genes based on KEGG database analysis. C: Top ten enriched pathways among downregulated genes based on KEGG database analysis. D: GSEA analysis of oxidative phosphorylation pathway based on global gene expression profiles. E: GSEA analysis of respiratory electron transport chain pathway based on global gene expression profiles. F: GSEA analysis of ferroptosis pathway based on global gene expression profiles. G, H: Measurement of oxygen consumption rate (OCR) changes in osteoblasts post-lipid droplet treatment using Seahorse metabolic assay. I, J: Flow cytometric detection of JC-1 staining in osteoblasts after lipid droplet exposure to evaluate mitochondrial membrane potential. K: Mitotracker fluorescence visualization of mitochondrial membrane potential changes in osteoblasts after lipid droplet treatment. L: Transmission electron microscopic examination of osteoblast mitochondrial morphology following lipid droplet treatment. M, N: Flow cytometry assessment of Annexin V and 7-AAD expression in osteoblasts post-lipid droplet treatment to quantify early apoptosis. O: Fluorescence observation of Bodipy-C11 expression in cells to monitor lipid peroxidation after lipid droplet treatment. P: Fluorescence visualization of intracellular Fe^2+^ levels indicating iron accumulation after lipid droplet treatment. Q, R: Western blot analysis of GPX4 and TFRC expression changes in osteoblasts following lipid droplet treatment, reflecting iron metabolism and oxidative stress responses.

In order to investigate the molecular underpinnings of lipid droplet-induced decrement in osteoblast function, we conducted high-throughput mRNA sequencing on lipid droplet-treated osteoblasts. This analysis revealed an alteration in gene expression, with 3,516 genes upregulated and 1,800 genes downregulated (Fig. 4A). Subsequent gene enrichment analysis identified the ferroptosis pathway as being upregulated, concomitant with a decrease in oxidative phosphorylation in osteoblasts exposed to lipid droplets (Fig. 4B, C). Further Gene Set Enrichment Analysis (GESA) revealed changes in the expression profiles associated with oxidative phosphorylation, the electron transport chain, and the ferroptosis pathway (Fig. 4D, E, F), suggesting a complex reprogramming of cellular metabolism and cell death mechanisms in response to lipid droplet accumulation. Given the importance of mitochondrial aerobic respiration for osteoblast maturation, we assessed the aerobic respiration capacity of lipid droplet-treated osteoblasts. Impairment in both basal and peak metabolic activity was observed in the lipid droplet-treated osteoblasts (Fig. 4G, H). Subsequent analyses employing various methods to evaluate mitochondrial functionality consistently indicated defective mitochondrial function in the osteoblasts exposed to lipid droplets (Fig. 4I–L). Furthermore, we noted an increased incidence of apoptosis in the lipid droplet-treated osteoblasts (Fig. 4M, N). To investigate the potential involvement of ferroptosis, we examined several ferroptosis-related markers and found elevated levels of lipid peroxidation (Fig. 4O) and increased intracellular iron content (Fig. 4P) in the osteoblasts treated with lipid droplets. These changes were accompanied by altered expression patterns of proteins closely associated with ferroptosis (Fig. 4Q, R), suggesting a possible role of ferroptosis in the observed functional deterioration of osteoblasts.

### Lipid droplet mediated regulation of ABHD5 in osteoblasts

**Fig. 5:**
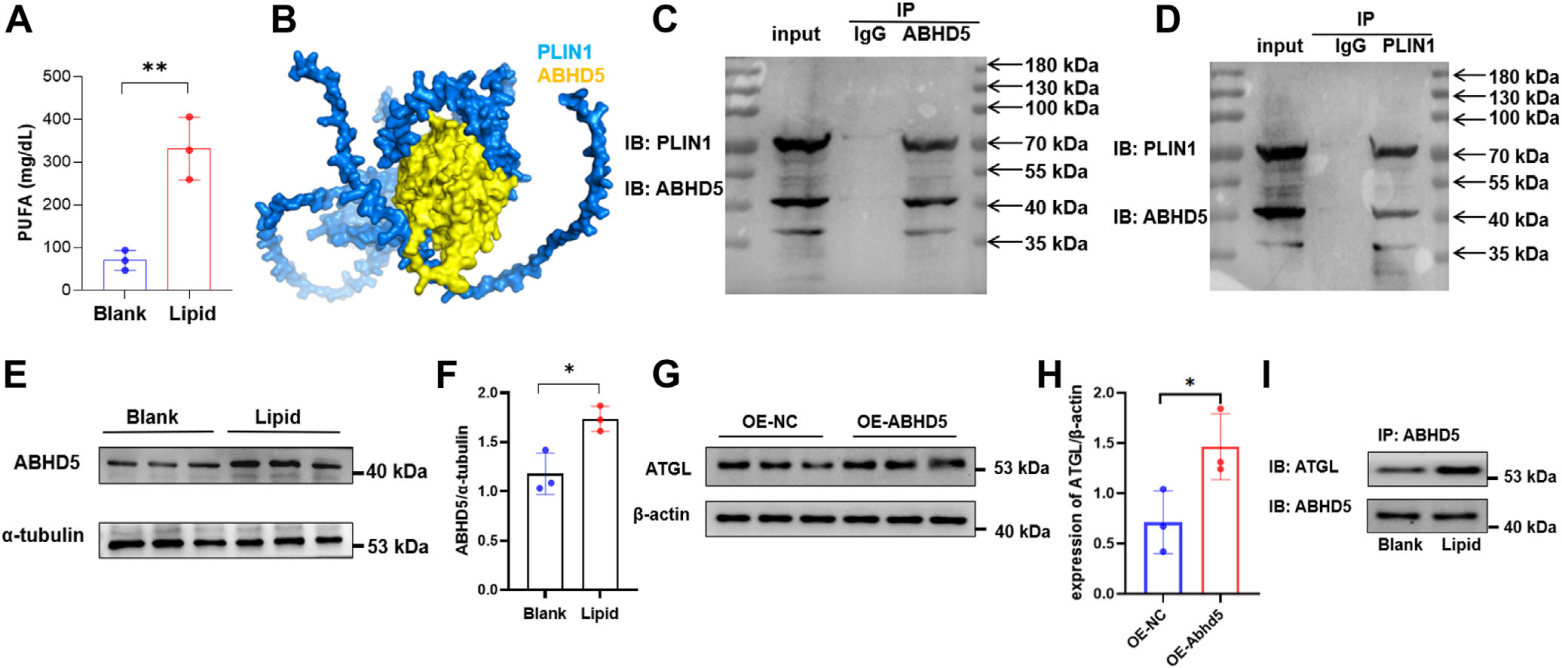
Lipid Droplet Treatment Augments ABHD5 and PUFA Expression in Osteoblasts. A: measurement of changes in PUFA levels within osteoblasts following lipid droplet treatment. B: molecular docking illustrating multiple binding sites between PLIN1 and ABHD5. C: co-immunoprecipitation (Co-IP) assay confirming the interaction between ABHD5 and PLIN1 after purifying ABHD5 protein. D: co-immunoprecipitation assay confirming the interaction between ABHD5 and PLIN1 after purifying PLIN1 protein. E, F: Western blot analysis determining the expression level of ABHD5 in osteoblasts after lipid droplet treatment. G and H: detecting ATGL expression levels using Western blotting after overexpressing ABHD5 in osteoblasts. I: after purifying the ABHD5 protein using immunoprecipitation, the expression levels of ATGL bound to equal amounts of ABHD5 were detected using Western blotting.

Emerging evidence implicated polyunsaturated fatty acids (PUFA) as key initiators of ferroptosis, and our observations in lipid droplet-treated osteoblasts support this notion (Fig. 5A). Given that lipid droplets were a major reservoir of unsaturated fatty acids, we hypothesized that the enhanced levels of these fatty acids within osteoblasts were associated with lipid droplet catabolism. Our sequencing data previously identified an upregulation of ABHD5, a recognized lipase activator, in osteoblasts treated with lipid droplets (Fig. 4A), prompting us to investigate potential interactions between lipid droplets and ABHD5. Molecular docking analyses identified multiple binding sites between PLIN1 and ABHD5 (Fig. 5B). Co-immunoprecipitation experiments confirmed the physical interaction between PLIN1 and ABHD5 (Fig. 5C, D), suggesting that this interaction may underlie the upregulation of ABHD5 in response to lipid droplet treatment (Fig. 5E). These findings underscore the dynamic interplay between lipid droplets and enzymatic regulators of lipid metabolism in osteoblastic contexts. We found that highly expressed ABHD5 can increase the expression levels of ATGL (Fig. 5G, H). Since ABHD5 interacts with ATGL to promote its activation, we tested the binding of ABHD5 to ATGL after lipid droplet treatment. Our results showed that after stimulation with lipid droplets, increased binding between ABHD5 and ATGL was found (Fig. 5I). This may explain the mechanism by which lipid droplet transfer to osteoblasts leads to lipolysis and an increase in PUFA.

### Ferro-1 restored osteoblast damage induced by lipid droplets and reverses bone loss in obese mice post-ovariectomy

**Fig. 6:**
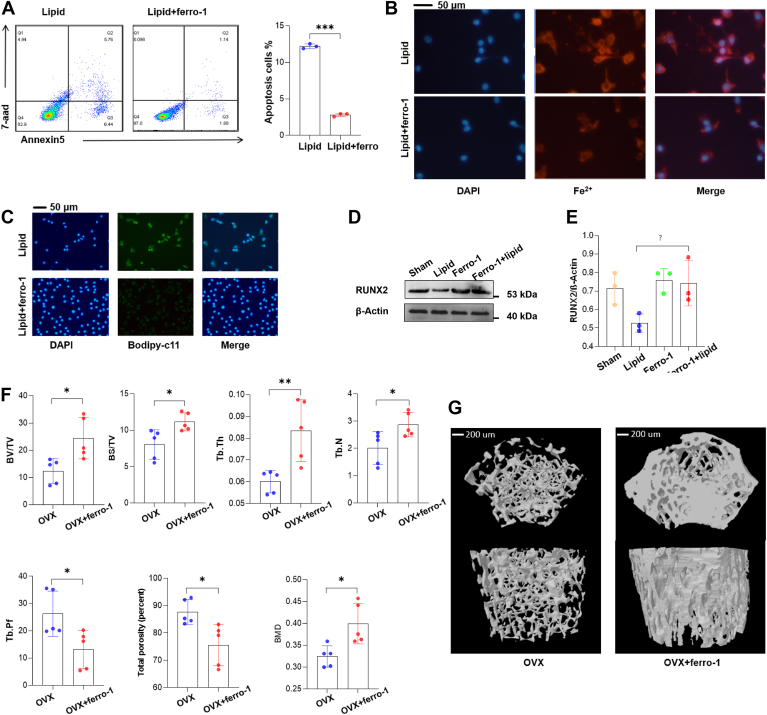
Ferroptosis Inhibitor Rescues Lipid Droplet-Induced Damage in Osteoblasts. A: Flow cytometric analysis of Annexin V and 7-AAD expression in lipid droplet-treated osteoblasts following incubation with the ferroptosis inhibitor Ferro-1 (1 μM) for 24 h. B: Fluorescence detection of Fe2+ levels in lipid droplet-treated osteoblasts after treatment with Ferro-1 (1 μM) for 24 h. C: Fluorescence measurement of Bodipy-C11 expression, indicative of lipid peroxidation, in lipid droplet-exposed osteoblasts treated with Ferro-1(1 μM) for 24 h. D and E: Western blot analysis of RUNX2 expression in osteoblasts with Ferro-1(1 μM) treatment for 24 h. F: quantification of trabecular bone parameters including Bone Volume/Tissue Volume (BV/TV), Bone Surface Area/Tissue Volume (BS/TV), Trabecular Thickness (Tb.Th), Trabecular Number (Tb.N), Bone Mineral Density (BMD), Trabecular Pattern Factor (Tb.Pf), and Total Porosity from microCT scans of the mouse femurs. G: micro-computed tomography (microCT) scanning of the femurs from osteoporotic mice, following administration of Ferro-1 (10 μM in 50 μl PBS). The administration of Ferro-1 was performed at a frequency of once per week, and this regimen was sustained for a period of two months.

Having elucidated the involvement of ferroptosis in the detrimental effects of lipid droplets on osteoblasts, we sought to ameliorate such damage using a ferroptosis inhibitor, ferrostatin-1 (ferro-1). *In vitro* experiments revealed that ferro-1 effectively blocked the death of osteoblasts induced by lipid droplets (Fig. 6A). Administration of ferro-1 led to a decrease in iron (Fe^2^⁺) accumulation (Fig. 6B) and lipid peroxidation (Fig. 6C) consequent to lipid droplet stress. Furthermore, ferro-1 treatment restored the impaired osteoblastic differentiation compromised by lipid droplets (Fig. 6D, E). Upon translating these in vitro findings to an in vivo scenario, we administered ferro-1 as a therapeutic agent in obese mice subjected to ovariectomy. Specifically, we attempted to model osteoporosis in obese mice and subsequently administered weekly intraperitoneal injections of ferro-1 for 2 months to observe its therapeutic effects on osteoporosis in these animals. We observed that ferro-1 treatment successfully reversed the bone mineral density loss and reduction seen in these post-surgical animals (Fig. 6F, G), thus substantiating its protective effect against osteoporosis-like conditions exacerbated by lipid droplet-induced ferroptosis.

**Fig. 7:**
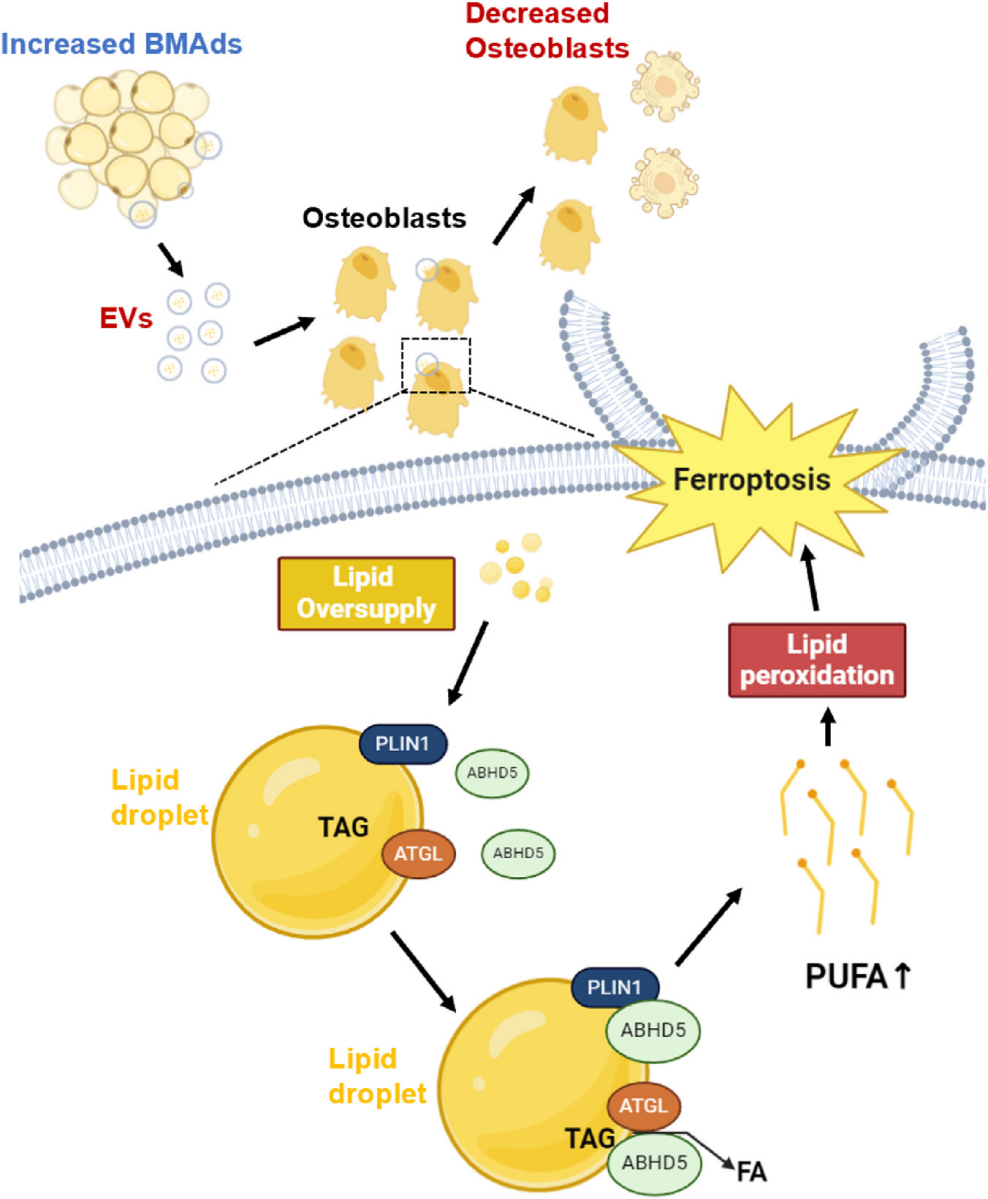
Scheme of the mechanism of this article. In conclusion, Extracellular vesicles derived from increased bone marrow adipocytes can transport lipid droplets into osteoblasts. The entry of lipid droplets into osteoblasts leads to upregulation of ABHD5 expression, activation of ATGL, promotion of lipolysis, and an increase in intracellular PUFA, resulting in an increased risk of ferroptosis in osteoblasts and a decrease in osteoblasts.

## Discussion

The prevailing assumption that higher body mass might protect against bone mineral depletion through enhanced mechanical loading has been challenged by emerging evidence (23, 24). Central to this paradoxical link between obesity and osteoporosis is the nature of adipose tissue, especially when excessively accumulated within the bone marrow (25). This ectopic lipid accumulation disrupts the delicate balance between osteoblast-mediated bone formation and osteoclastic bone resorption, ultimately resulting in a net loss of bone mass (26). Marrow-residing adipocytes dynamically regulate skeletal metabolism by secreting a variety of bioactive molecules, including cytokines (eg, leptin, adiponectin) (27, 28), influencing the functional interplay among neighboring cellular populations. Leptin, for instance, promotes osteoblast differentiation while inhibiting osteoclastogenesis; however, obesity-induced leptin resistance disrupts this regulatory balance (29). Furthermore, bone marrow adipocytes contribute to a chronic, low-intensity inflammatory milieu within osseous tissue, impeding osteoblast function and stimulating osteoclastic activity, accelerating bone degradation (30). These multidimensional impacts underscore the pivotal role of marrow adiposity in osteoporosis pathophysiology, necessitating a deeper understanding of the molecular dialogues orchestrating adipocyte-bone cell interactions.

The central focus of our investigation was to investigate the specific mechanisms through which lipid droplets derived from BMAds affect osteoblast function and contribute to osteoporosis pathophysiology. It has been reported that adipocytes can secrete vesicles in response to variable metabolic conditions (31). For instance, lipolytic signals, and cellular stressors such as DNA damage would increase the secretion of EVs derived from adipocytes in a *P*53-dependent manner (32). Besides, obesity was associated with an elevated secretion of vesicles specifically from adipocytes (31, 32). Based on the clinical findings as well as previous literature, our hypothesis centered on the idea that these lipid droplets might be transferred to osteoblasts via sEVs, which may be simulated by various factors such as obesity and lipolysis, and thus influence the osteoblast behavior through a cascade involving lipolytic regulators and subsequent cellular stress responses. Our findings supported this hypothesis. Our results also demonstrated that the transfer of lipid droplets leads to upregulation of ABHD5, a critical lipolytic mediator (33, 34), resulting in the accumulation of polyunsaturated fatty acids (PUFAs) and triggering ferroptosis in osteoblasts (35, 36, 37).

By identifying ABHD5 as a key player and linking it to ferroptotic cell death, our study provides a new perspective on the detrimental impact of lipid overload on bone health. The observed correlation between lipid droplet-induced ABHD5 activation and subsequent accumulation of PUFAs mechanistically explains the decline in osteoblast viability and differentiation, aligning with our initial hypotheses. Additionally, the discovery of a non-canonical lipid droplet transmission pathway via sEVs corresponds to a previous study that demonstrated a new dimension to cell-cell communication in the bone microenvironment, enriching our understanding of metabolic crosstalk (38, 39). These results significantly advance our understanding of the complex relationship between bone metabolism and adiposity.

Recently, an increasing number of researchers have studied the relationship between ferroptosis and osteoporosis. In osteoblasts, ferroptosis is associated with oxidative stress and iron overload, leading to decreased bone formation. This process involves the reduction of crucial antioxidant systems, such as GPX4, and the accumulation of reactive oxygen species (ROS), which damage cellular components and impair osteoblast function (40). Metabolic disorders such as type 2 diabetes have been reported to be associated with ferroptosis in osteoblasts, leading to bone loss. Cells in high-fat or high-glucose environments are prone to ferroptosis (41, 42). Zhe *et al.* applied mice experiments and proved that a high-fat diet could increase bone loss by inducing ferroptosis in osteoblasts (43). Chen *et al.* found that HFD-induced obese rats exhibited decreased levels of ferroptosis inhibitory protein (44). Wang *et al.* found that ED-71 could activate the HIF1α pathway and relieve the ferroptosis induced by high glucose. (45) However, the research on the relationship between ferroptosis and osteoporosis is still in its relatively early stages, and the specific mechanisms, key molecules as well as involved signaling pathways remain to be explored. Our study advances the field by highlighting the role of ABHD5 and its involvement in ferroptosis, shifting the focus from lipid accumulation to the active role of lipid metabolism in determining cell fate, which is a previously underappreciated aspect of bone biology. The mechanism and pathway found in the current paper could help to explain the potential association between obesity, high-fat diet, and osteoporosis. However, it should be admitted that ferroptosis may also occur in osteoclasts and reduce bone resorption. A strategy that could regulate the ferroptosis of osteoblasts and osteoclasts may provide potential treatment with regard to osteoporosis.

Despite these advancements, our study has limitations. The use of in vitro and murine models, while informative, may not fully replicate human osteoporosis complexities. Human-based in vitro models and larger-scale clinical studies are needed for validation and to explore potential population variations. Moreover, expanding sample sizes would enhance statistical power and generalizability. Future studies should address confounding factors, such as bone marrow adipocyte heterogeneity and individual metabolic profiles. Also, the upstream mechanism that regulates the BMAds to release sEVs containing lipid droplets still remains to be further studied in future research.

Looking ahead, numerous avenues exist to advance our understanding and translation into clinical practice. Detailed investigation of the spatiotemporal dynamics governing lipid droplet journey and ABHD5 activation mechanisms is crucial. Uncovering these details may reveal novel therapeutic targets for modulating lipid-induced effects on osteoblasts. Characterizing the sEV-mediated lipid transfer process, including cargo, receptor-ligand interactions, and signaling cascades, could inform targeted interventions to restore lipid balance and prevent ferroptosis, ultimately preserving or enhancing bone mass and strength. Translating these insights into clinically viable biomarkers and therapies requires multidisciplinary efforts integrating fundamental biology, advanced technology, and clinical application. Developing strategies to interfere with sEV-mediated lipid transfer holds promise for innovative osteoporosis and obesity-related bone disease treatments.

## Conclusion

In conclusion, our study has revealed a previously unrecognized link between BMAds and bone loss, involving ferroptosis. We found that these abnormal lipid droplets may originate from bone marrow adipocytes through exosome delivery. Upon entry into osteoblasts, the expression of ABHD5 is upregulated, which is a crucial activator of intracellular lipolysis. Subsequently, this leads to an upregulation of lipolysis and an increase in intracellular PUFA. Consequently, this induces ferroptosis in osteoblasts, ultimately leading to osteoporosis. Our findings emphasize the role of lipid droplets derived from BMAds in affecting the health of osteoblasts. Despite the study's limitations, these findings set the stage for future research that could enhance our understanding of bone health issues and help create targeted treatments.

## Data availability

The data that support the findings of this study are available from the corresponding author, upon reasonable request.

## Supplemental data

This article contains supplemental data.

## Conflicts of interest

The author(s) have no conflicts of interest relevant to this article.

## References

[1] Nandy A., Rendina-Ruedy E. (2021). Bone marrow adipocytes - good, bad, or just different. Best Pract. Res. Clin. Endocrinol. Metab..

[2] Scheller E.L., Doucette C.R., Learman B.S., Cawthorn W.P., Khandaker S., Schell B. (2015). Region-specific variation in the properties of skeletal adipocytes reveals regulated and constitutive marrow adipose tissues. Nat. Commun..

[3] Abuna R.P.F., Almeida L.O., Souza A.T.P., Fernandes R.R., Sverzut T.F.V., Rosa A.L. (2021). Osteoporosis and osteoblasts cocultured with adipocytes inhibit osteoblast differentiation by downregulating histone acetylation. J. Cell Physiol..

[4] Hu K., Deya Edelen E., Zhuo W., Khan A., Orbegoso J., Greenfield L. (2023). Understanding the consequences of fatty bone and fatty muscle: how the osteosarcopenic adiposity phenotype uncovers the deterioration of body composition. Metabolites.

[5] Cai G.P., Liu Y.L., Luo L.P., Xiao Y., Jiang T.J., Yuan J. (2022). Alkbh1-mediated DNA N6-methyladenine modification regulates bone marrow mesenchymal stem cell fate during skeletal aging. Cell Prolif..

[6] Feng S., Feng Z., Wei Y., Zheng X., Deng Z., Liao Z. (2024). EEF1B2 regulates bone marrow-derived mesenchymal stem cells bone-fat balance via Wnt/β-catenin signaling. Cell Mol. Life Sci..

[7] Milišić L., Vegar-Zubović S., Valjevac A. (2020). Bone marrow adiposity is inversely associated with bone mineral density in postmenopausal females. Med. Glas. (Zenica).

[8] Wong A.K., Chandrakumar A., Whyte R., Reitsma S., Gillick H., Pokhoy A. (2020). Bone marrow and muscle fat infiltration are correlated among postmenopausal women with osteoporosis: the AMBERS cohort study. J. Bone Mineral Res..

[9] Cawthorn W.P., Scheller E.L., Learman B.S., Parlee S.D., Simon B.R., Mori H. (2014). Bone marrow adipose tissue is an endocrine organ that contributes to increased circulating adiponectin during caloric restriction. Cell Metab..

[10] Yue R., Zhou B.O., Shimada I.S., Zhao Z., Morrison S.J. (2016). Leptin receptor promotes adipogenesis and reduces osteogenesis by regulating mesenchymal stromal cells in adult bone marrow. Cell Stem Cell.

[11] Li Z., Bowers E., Zhu J., Yu H., Hardij J., Bagchi D.P. (2022). Lipolysis of bone marrow adipocytes is required to fuel bone and the marrow niche during energy deficits. eLife.

[12] van Niel G., Carter D.R.F., Clayton A., Lambert D.W., Raposo G., Vader P. (2022). Challenges and directions in studying cell-cell communication by extracellular vesicles. Nat. Rev. Mol. Cell Biol..

[13] Bonavina G., Mamillapalli R., Krikun G., Zhou Y., Gawde N., Taylor H.S. (2024). Bone marrow mesenchymal stem cell-derived exosomes shuttle microRNAs to endometrial stromal fibroblasts that promote tissue proliferation/regeneration/and inhibit differentiation. Stem Cell Res. Ther..

[14] Wang S., Jia Z., Dai M., Feng X., Tang C., Liu L. (2024). Advances in natural and synthetic macromolecules with stem cells and extracellular vesicles for orthopedic disease treatment. Int. J. Biol. Macromol..

[15] Lin L., Guo Z., He E., Long X., Wang D., Zhang Y. (2023). SIRT2 regulates extracellular vesicle-mediated liver-bone communication. Nat. Metab..

[16] Zhang Y., Zhang C., Wang J., Liu H., Wang M. (2021). Bone-adipose tissue crosstalk: role of adipose tissue derived extracellular vesicles in bone diseases. J. Cell Physiol..

[17] König T., McBride H.M. (2024). Mitochondrial-derived vesicles in metabolism, disease, and aging. Cell Metab..

[18] Wang Z.X., Lin X., Cao J., Liu Y.W., Luo Z.W., Rao S.S. (2024). Young osteocyte-derived extracellular vesicles facilitate osteogenesis by transferring tropomyosin-1. J. Nanobiotechnol..

[19] Zhang X., Yang J., Ma S., Gao X., Wang G., Sun Y. (2024). Functional diversity of apoptotic vesicle subpopulations from bone marrow mesenchymal stem cells in tissue regeneration. J. Extracell. Vesicles.

[20] Flaherty S.E., Grijalva A., Xu X., Ables E., Nomani A., Ferrante A.W. (2019). A lipase-independent pathway of lipid release and immune modulation by adipocytes. Science.

[21] Maurel D.B., Pallu S., Jaffré C., Fazzalari N.L., Boisseau N., Uzbekov R. (2012). Osteocyte apoptosis and lipid infiltration as mechanisms of alcohol-induced bone loss. Alcohol Alcohol..

[22] Al Saedi A., Debruin D.A., Hayes A., Hamrick M. (2022). Lipid metabolism in sarcopenia. Bone.

[23] Dorilleau C., Kanagaratnam L., Charlot I., Hittinger A., Bertin E., Salmon J.H. (2024). The least significant change on bone mineral density scan increased in patients with higher degrees of obesity. Aging Clin. Exp. Res..

[24] Chiu C.T., Lee J.I., Lu C.C., Huang S.P., Chen S.C., Geng J.H. (2024). The association between body mass index and osteoporosis in a Taiwanese population: a cross-sectional and longitudinal study. Sci. Rep..

[25] Wang T., Zhao C., Zhang J., Li S., Zhang Y., Gong Y. (2024). Whitening of brown adipose tissue inhibits osteogenic differentiation via secretion of S100A8/A9. iScience.

[26] Li J., Lu L., Liu L., Wang C., Xie Y., Li H. (2024). The unique role of bone marrow adipose tissue in ovariectomy-induced bone loss in mice. Endocrine.

[27] Deepika F., Bathina S., Armamento-Villareal R. (2023). Novel adipokines and their role in bone metabolism: a narrative review. Biomedicines.

[28] Inoue K., Qin Y., Xia Y., Han J., Yuan R., Sun J. (2023). Bone marrow Adipoq-lineage progenitors are a major cellular source of M-CSF that dominates bone marrow macrophage development, osteoclastogenesis, and bone mass. eLife.

[29] Patil J.D., Fredericks S. (2024). The role of adipokines in osteoporosis management: a mini review. Front. Endocrinol..

[30] Li J., Zou Z., Su X., Xu P., Du H., Li Y. (2024). Cistanche deserticola improves ovariectomized-induced osteoporosis mainly by regulating lipid metabolism: insights from serum metabolomics using UPLC/Q-TOF-MS. J. Ethnopharmacol..

[31] Roca-Rivada A., Alonso J., Al-Massadi O., Castelao C., Peinado J.R., Seoane L.M. (2011). Secretome analysis of rat adipose tissues shows location-specific roles for each depot type. J. Proteomics.

[32] Huang Y., Hertzel A.V., Fish S.R., Halley C.L., Bohm E.K., Martinez H.M. (2023). TP53/p53 facilitates stress-induced exosome and protein secretion by adipocytes. Diabetes.

[33] Yang Y., Peng Y., Yu B., Wang H. (2024). ABHD5-CPT1B: an important way of regulating placental lipid metabolism in gestational diabetes mellitus. Arch. Med. Res..

[34] Corvillo F., Abel B.S., López-Lera A., Ceccarini G., Magno S., Santini F. (2023). Characterization and clinical association of autoantibodies against Perilipin 1 in patients with acquired generalized lipodystrophy. Diabetes.

[35] Morgan P.K., Pernes G., Huynh K., Giles C., Paul S., Smith A.A.T. (2024). A lipid atlas of human and mouse immune cells provides insights into ferroptosis susceptibility. Nat. Cell Biol..

[36] Liu X., Chen Z., Yan Y., Zandkarimi F., Nie L., Li Q. (2024). Proteomic analysis of ferroptosis pathways reveals a role of CEPT1 in suppressing ferroptosis. Protein Cell.

[37] Suda A., Umaru B.A., Yamamoto Y., Shima H., Saiki Y., Pan Y. (2024). Polyunsaturated fatty acids-induced ferroptosis suppresses pancreatic cancer growth. Sci. Rep..

[38] Liu Y., Ren L., Li M., Zheng B., Liu Y. (2024). The effects of hypoxia-preconditioned dental stem cell-derived secretome on tissue regeneration. Tissue Eng. Part B Rev..

[39] Chen Z., Zhou T., Luo H., Wang Z., Wang Q., Shi R. (2024). HWJMSC-EVs promote cartilage regeneration and repair via the ITGB1/TGF-β/Smad2/3 axis mediated by microfractures. J. Nanobiotechnol..

[40] Liu P., Wang W., Li Z., Li Y., Yu X., Tu J. (2022). Ferroptosis: a new regulatory mechanism in osteoporosis. Oxidative Med. Cell Longev..

[41] Turchi R., Tortolici F., Guidobaldi G., Iacovelli F., Falconi M., Rufini S. (2020). Frataxin deficiency induces lipid accumulation and affects thermogenesis in brown adipose tissue. Cell Death Dis..

[42] Ma W.Q., Sun X.J., Zhu Y., Liu N.F. (2021). Metformin attenuates hyperlipidaemia-associated vascular calcification through anti-ferroptotic effects. Free Radic. Biol. Med..

[43] Zhu R., Wang Z., Xu Y., Wan H., Zhang X., Song M. (2022). High-fat diet increases bone loss by inducing ferroptosis in osteoblasts. Stem Cell Int..

[44] Chen X., Liu C., Yu R., Gan Z., Zhang Z., Chen Z. (2023). Interaction between ferroptosis and TNF-α: impact in obesity-related osteoporosis. FASEB J..

[45] Wang M., Liu Y., Gui H., Ma G., Li B., Zhang Z. (2024). ED-71 ameliorates bone regeneration in type 2 diabetes by reducing ferroptosis in osteoblasts via the HIF1α pathway. Eur. J. Pharmacol..

