# Supplementary material for: sEV-mediated lipid droplets transferred from bone marrow adipocytes promote ferroptosis and impair osteoblast function
Source: J Lipid Res. 2024 Sep 24;65(11):100657. doi: 10.1016/j.jlr.2024.100657 (PMC11535364; doi:10.1016/j.jlr.2024.100657)
Supplement: Supplemental data [file mmc1.docx]

**Supplementary file 1. Clinicodemographic characteristics and bone mineral density evaluation of all study patients**

|  | Normal  (n=159) | Osteoporosis  (n=36) | p value |
| --- | --- | --- | --- |
| Characteristics |  |  |  |
| Age | 59.05±0.86 | 67.22±1.42 | <0.001 |
| BMI | 25.98±0.26 | 24.25±0.53 | 0.005 |
| Ethnicities  East Asian | 159 (100%) | 36 (100%) | 1.000 |
| Gender |  |  | <0.001 |
| Male | 89(56.0%) | 7(19.4%) |  |
| Female | 70(44.0%) | 29(80.6%) |  |
| Diabetes |  |  | <0.001 |
| Yes | 18(11.3%) | 3(8.3%) |  |
| No | 141(88.7%) | 33(91.7%) |  |
| Cigarette |  |  | 0.560 |
| Yes | 8(5.0%) | 1(2.8%) |  |
| No | 151(95.0%) | 35(97.2%) |  |
| DEXA T-Score |  |  |  |
| L1-L4 | 0.27±0.12 | -2.54±0.17 | <0.001 |
| Femoral neck | -0.20±0.05 | -2.83±0.05 | <0.001 |
| Total | 0.38±0.06 | -2.25±0.09 | <0.001 |
| FFA  LDL-C  HDL-C  TG  TC  APO A1  APO B  APO E | 0.58±0.26  2.96±0.89  1.18±0.31  2.02±1.31  5.28±1.11  1.31±0.21  0.84±0.22  38.78±25.27 | 0.69±0.27  3.25±1.08  1.24±0.32  1.54±0.90  5.45±1.30  1.35±0.23  0.88±0.27  42.17±17.64 | 0.021  0.095  0.302  0.037  0.432  0.287  0.356  0.447 |
